# Dentistry in Great Britain

**Published:** 1859-06

**Authors:** 


### Selections.

### DENTISTRY IN GREAT BRITAIN.

*Severe forms of disease arising from retention of Decayed Teeth.* By Mr. Clendon, Surgeon-Dentist to the Westminster Hospital. (*British Medical Journal*, June, 1858.)—After describing very graphically the ordinary effects of diseased teeth, Mr. Clendon proceeds to speak of the more extraordinary effects—disease of antrum, fungoid tumors of the gums, facial neuralgia, facial paralysis, and spasms in certain forms, etc. These remarks are illustrated by cases, and of these we take one as well worthy of notice.—*Lanc. & Obs.*

*Case 7.—Abscess and Paralysis of the Face.*—R. W.——, æt. 34, harness-maker, gave me the following history: "In January last (1857), I felt pain in the ear, which began to discharge; then an abscess formed in front of the ear; this was lanced at the Northern Dispensary, and it continued to discharge freely for a month or more. Having lost the hearing on that side, I went to the Ear Infirmary in Soho Square for two months, without any benefit. Discharge of the abscess through the ear continued until December. I then first began to feel severe pain when I attempted to move the jaw; this was followed by inability to close the eye, or to put out my tongue. In that state I came to this (the Westminster) hospital, in January last, and was admitted under the care of Dr. Reynolds."

From the constant pain and tenderness on pressure, Dr. Reynolds, suspecting some local cause of irritation, sent him to me, in order that his mouth might be carefully examined. When I first saw him, there was great difficulty in opening his mouth; severe pain in the condyle when he attempted to move the lower jaw, and on pressure being made in that direction; he was unable to close the eye-lids, to contract the orbicular muscle of the eye, to inflate the cheek, or to protrude the tongue beyond the front teeth, gasping when he endeavored to do it. On attempting to whistle, the mouth and nostrils were drawn to the opposite side; the external meatus of the ear was so contracted, that a fine probe only would pass; and through the orifice, on pressure being made on the cheek, pus oozed out. He had no toothache, nor decayed tooth, that he knew of; but I found the teeth on the affected side incrusted with tartar, from want of use. This to me is always a suspicious circumstance. He attributed it to pain in the hinge of the jaw in eating, and to his inability to remove the food from the cheek, owing to the paralysis of the muscles. On passing a curved instrument under the gum between two molar teeth, he felt some pain in one tooth, and, repeating the experiment with the same result, I determined to remove it. There was a cavity in it, quite out of sight, and almost out of reach, through which, I have no doubt, air had from the first freely passed, and given rise to all the mischief. On washing out the mouth, he at once expressed a sense of relief; found he could open his mouth easily; and to the surprise of all present, he could protrude his tongue to the full extent without any apparent effort, and also partially close the eye. The relief might be described as instantaneous. The inability to contract the muscles of the mouth, to inflate both cheeks, and to raise the angle of the mouth, still continues; and, although it is asserted that in the severest lesions the nerves do not slough, I nevertheless expect some branches of the portio dura have been destroyed in the long-continued abscess, and that loss of power in some of the muscles, as well as of hearing, on that side, are permanent and irremediable.

The paper concludes with the following practical remarks:

"Now, in all these cases, whatever the symptoms, and whether the pain be severe, mitigated or altogether absent, there is throughout but one indication—namely, an effort of nature to get rid of the offending body, which we, if we would endeaver wisely to assist and second, instead of to counteract her, ought at once to seek out and remove.

"But here is the difficulty. The patient has a dread of the operation, and will run all risks, submit to any course of treatment, or any amount of suffering, rather than undergo it. An old lady of seventy once told me that, from her earliest recollection down to the period when the last remaining tooth had worked its own way out, she had seldom been free from pain for a month together; and yet she could never summon courage to submit to the operation of extraction. In her own emphatic words, 'she felt she must die first.' But in severe cases, where there is great tenderness or pain, or where the operation would be more than usually painful and difficult to perform, chloroform will deprive the patient of all excuse, and prove a great blessing. It allows the practitioner to open and examine the mouth carefully, perhaps for the first time, and then, at his leisure, to seek for and remove all that he deems necessary. It is true that, owing to some few casualties, which from the first I anticipated, some persons have as much dread of chloroform as of the operation itself; but my experience in some 3,000 cases, taken indiscriminately, and extending over a period of more ten years, satisfies me that, although, like all powerful agents, it is dangerous if misused, it can always be given with safety if administered with proper care. I can truly aver that, until the other day, when, in a case of protracted operation at the shoulder-joint, much blood being lost, syncope came on while the patient was fully under the influence of the chloroform, but which was detected and counteracted on the instant, I have not had one case which, during its administration or subsequently, ever gave me a moment's uneasiness.

"Often, however, there is another difficulty; and that is on the part of the practitioner. Perhaps he examines the mouth, and ascertains the cause of the pain to be the root of a tooth broken off and buried deep in the gum, or he finds a number of roots clustered together, presenting no tangible surface for the grasp of an instrument, and difficult to reach, even with an elevator. He sees they ought to be removed, but shrinks from the task; to use a familiar phrase, 'he does not like the looks of them;' and, therefore, recommends some palliative mode of treatment, and patience. In point of fact, from not feeling quite equal to the emergency, or wanting confidence in the use of the instrument, he is reluctantly compelled to leave his patient to an indefinite period of suffering, and perhaps to some of the evil results I have already pointed out. This abnegation of his legitimate functions, arising purely from want of confidence in himself, has notoriously had the effect of driving this department of surgery into the hands of unqualified and often uneducated practitioners, styling themselves *surgeon*-dentists, and affecting to consider the mouth as their own legitimate domain, to which the surgeon has no claim. But, whatever the cause, the result is the same; for now the old story is repeated: the cheek swells, pus forms, and there is either a circumscribed or diffused abscess. This is poulticed, to promote absorption, or to assist resolution, as the case may be. Now, I can not too forcibly insist that fomenting the cheek under such circumstances is the most erroneous step of all; for, should the abscess point outwardly, there will be a sinus from which pus may continue to flow, perhaps for a year or more, as long as there is any root or carious bone to exfoliate, leaving, when healed, a deep pit or cicatrix in the cheek, which disfigures the patient for life. This is not a pleasant alternative for a man, but a very serious drawback to a woman, in the preservation of whose good looks we all feel a natural interest. The tooth should be removed, when the pus will immediately flow through the aperture made; or, failing that, hot water, or hot bread and water fomentations, should be used *in* the mouth; and, as soon as possible, the abscess, however deeply seated, should be opened freely through the mucous membrane, and the pus allowed to escape *into the mouth.*

"In conclusion, I think that no one will deny that it should be a part of a medical man's education to know how to deal with such cases; and I trust that all you who have kindly gone with me through these observations will have felt their interest and importance; and, seeing how much suffering and mischief may spring from so small a cause as a diseased tooth, you will sympathise in my earnest wish to obtain the diffusion of more enlightened views on this much neglected, and therefore little understood, department of surgery. Every day confirms my experience of its necessity, and strengthens my desire to see it accomplished. Many complaints that come under the notice of the practitioner as diseases of the body, are, in reality, diseases of the teeth. It is clearly impossible to treat the body as a whole, if we are ignorant of its parts; and I will venture to say there is no part much more widely or universally affecting the general system than that of the teeth."—*Ranking's Abstract.*

